# Suppression of Pitch Labeling: No Evidence for an Impact of Absolute Pitch on Behavioral and Neurophysiological Measures of Cognitive Inhibition in an Auditory Go/Nogo Task

**DOI:** 10.3389/fnhum.2020.585505

**Published:** 2020-11-12

**Authors:** Marielle Greber, Lutz Jäncke

**Affiliations:** ^1^Division Neuropsychology, Department of Psychology, University of Zurich, Zurich, Switzerland; ^2^University Research Priority Program (URPP), Dynamics of Healthy Aging, University of Zurich, Zurich, Switzerland

**Keywords:** absolute pitch (AP), Go/Nogo, musicians, auditory, inhibition, event-related potential (ERP)

## Abstract

Pitch labeling in absolute pitch (AP), the ability to recognize the pitch class of a sound without an external reference, is effortless, fast, and presumably automatic. Previous studies have shown that pitch labeling in AP can interfere with task demands. In the current study, we used a cued auditory Go/Nogo task requiring same/different decisions to investigate both behavioral and electrophysiological correlates of increased inhibitory demands related to automatic pitch labeling. The task comprised two Nogo conditions: a Nogo condition with pitch differences larger than one semitone, and a second Nogo condition with pitch differences of only a quarter semitone. The first Nogo condition tested if auditory-related inhibition processes are generally altered in AP musicians. The second Nogo condition tested the suppressibility of the pitch labeling using a Stroop-like effect: the two tones belonged to the same pitch class but were not identical in terms of tone frequency. If pitch labeling cannot be suppressed, the conflicting information would be expected to increase the inhibitory load in AP musicians. Our data provided no evidence for an increased difficulty to inhibit a prepotent response or to suppress conflicting pitch-labeling information in AP: AP musicians showed similar commission error rates as non-AP musicians in both Nogo conditions. N2d and P3d amplitudes of AP musicians were also comparable to those of non-AP musicians. The event-related potentials (ERPs) were, however, modulated by the Nogo condition, probably indicating an effect of stimulus similarity. It is possible that, depending on the context, pitch labeling in AP musicians is not entirely automatic and can be suppressed.

## Introduction

Pitch is one of the main perceptual properties of musical tones. Most people perceive pitch not in absolute but rather in relative terms, i.e., they register whether a pitch is higher or lower compared to a previous pitch. Professional musicians are further trained to determine the exact amount of the relative difference between two pitches in terms of musical intervals. Using this so-called relative-pitch (RP) ability, most musicians can reconstruct the pitch of a tone when presented with a reference tone. Only about 0.01% of the general population (Bachem, 1955; Profita and Bidder, 1988; Takeuchi and Hulse, 1993) and about 4–15% of musicians (Baharloo et al., 1998; Gregersen et al., 1999, 2001; Leite et al., 2016) possess the unique ability to recognize the pitch class of a tone or to produce a specific pitch without the aid of a reference tone. This ability is referred to as absolute pitch (AP; Deutsch, 2013).

Pitch identification in AP is fast and effortless (Miyazaki, 1990; Deutsch, 2013), and is even presumed to be automatic (Levitin and Rogers, 2005). The extent of this automaticity has been studied primarily using auditory Stroop tasks (Miyazaki, 2004; Itoh et al., 2005; Hsieh and Saberi, 2008; Akiva-Kabiri and Henik, 2012; Schulze et al., 2013). Originally, the Stroop effect (Stroop, 1935) describes the phenomenon that naming the ink color of a semantically incongruent color word (e.g., the word “RED” printed in the color blue) is slower than naming the ink color of solid-color squares. By contrast, the latency for reading the words printed in color is not reliably increased compared to the same words printed in black. The more automatic process (i.e., reading) impedes the less automatic process (i.e., color naming) but not vice versa. Stroop tasks (for an overview, see MacLeod, 1991) use this asymmetrical effect to assess the ability to inhibit cognitive interference. In AP research, auditory analogs of the Stroop task typically consist of trials where the pitch of a tone is either congruent or incongruent with the sung tone label (integrated stimuli; e.g., Itoh et al., 2005) or a visual note presented simultaneously (non-integrated stimuli; e.g., Akiva-Kabiri and Henik, 2012). In incongruent trials, AP musicians consistently show increased response times for label/note naming compared to congruent trials. Pitch labeling, on the other hand, seems to be less affected by incongruence (Akiva-Kabiri and Henik, 2012). Furthermore, studies have shown that AP musicians perform worse than non-AP musicians in interval identification when given an out-of-tune context (Miyazaki, 1992, 1993) and in recognition of transposed atonal melodies (Miyazaki and Rakowski, 2002). As Dooley and Deutsch (2010, 2011) pointed out, these findings may reflect Stroop-like interference effects rather than a general disadvantage in relative-pitch tasks. Taken together, this suggests that the pitch-labeling process in AP is highly automatic and difficult to suppress.

At a more general level, several neurophysiological studies reported that AP musicians showed increased activity in different brain areas (e.g., in the auditory cortex, the planum temporale, the inferior frontal gyrus, and the DLPFC) during acoustic stimulation compared to non-AP musicians even when not instructed to perform pitch labeling (Zatorre et al., 1998; Ohnishi et al., 2001; Wu et al., 2008; Wengenroth et al., 2014; Burkhard et al., 2019; Leipold et al., 2019a). These findings indicate that tone processing in AP musicians is generally altered and that at least some AP-specific processes might be automatically triggered by musical tones. This assumption received further support from a recent decoding study that found a greater representational similarity in electrophysiological activity between a pure listening task and a labeling task in AP musicians compared to non-AP musicians (Leipold et al., 2019b).

The current study aimed to further explore the automaticity and suppressibility of pitch labeling in AP by examining both electrophysiological and behavioral correlates of another prominent psychological paradigm: the Go/Nogo task. Like Stroop tasks, Go/Nogo tasks are widely used to evaluate executive functions, particularly the capacity for inhibitory control. Typically, participants are instructed to press a button as quickly as possible whenever a target stimulus appears within a series of stimuli (Go) and to withhold the button press when a non-target stimulus appears (Nogo). A prominent advantage of Go/Nogo tasks is that the cognitive processes can be evaluated by both behavioral and well-established electrophysiological measures. The main behavioral measures are failures to inhibit a prepared motor response in Nogo trials (called commission errors or false alarms), failures to respond to the target in Go trials (called omission errors or misses), and response times in Go trials. The main electrophysiological measures are two event-related potential (ERP) components associated with reactive cognitive control: the Nogo-N2 and the Nogo-P3. The Nogo-N2, a negative deflection at frontal-midline sites, peaks around 200–400 ms after stimulus onset. The Nogo-P3, the subsequent frontocentral positive shift, is at its maximum about 300–600 ms after stimulus onset (e.g., Pfefferbaum et al., 1985; Pfefferbaum and Ford, 1988; Jodo and Kayama, 1992; Falkenstein et al., 1995, 1999; Bokura et al., 2001; Folstein and Van Petten, 2008; Gajewski and Falkenstein, 2013). Both the N2 and the P3 are usually evaluated by subtracting the Go ERP from the Nogo ERP (Nogo minus Go). In the following, we will refer to the N2 and P3 of the resulting difference wave as N2d and P3d. The exact cognitive subprocesses of response inhibition reflected by the N2 and P3 have been controversially discussed (for a review, see Huster et al., 2013). While some studies associated the N2 with pre-motor inhibitory processes (Jodo and Kayama, 1992; Falkenstein et al., 1999; Bokura et al., 2001; Gajewski and Falkenstein, 2013), other research indicates that the N2 reflects response activation (Bruin et al., 2001) or conflict monitoring (Nieuwenhuis et al., 2003; Donkers and Van Boxtel, 2004; Yeung et al., 2004; Enriquez-Geppert et al., 2010; Kropotov et al., 2017). The P3 has been suggested to mirror inhibitory processes or the evaluation of successful inhibition (Bokura et al., 2001; Bruin and Wijers, 2002; Donkers and Van Boxtel, 2004; Smith et al., 2008; Enriquez-Geppert et al., 2010; Albert et al., 2013; Kropotov et al., 2017).

In the current study, 54 AP and 51 non-AP musicians completed a cued (two-stimulus) Go/Nogo task with acoustic stimuli (i.e., piano tones and environmental sounds). The cue (i.e., a piano tone) was used to establish a prepotent tendency to respond. A button press was required whenever two identical piano tones were presented in succession (Go condition). In trials where two non-identical piano tones were presented, the button press had to be withheld (Nogo condition). Two variations of the Nogo condition were included. In the first Nogo condition, the two presented piano tones differed by at least one semitone (100–700 cents). In the second Nogo condition, the two piano tones differed by only a quarter semitone (25 cents). Using these two Nogo conditions allowed us to study different aspects of pitch processing in AP: (1) inhibition of a possibly stronger neurophysiological activation induced by tones; and (2) suppressibility of pitch labeling. As described above, acoustic stimulation elicits strong neurophysiological activation in AP musicians. This, in turn, might influence subsequent cognitive processes and their respective neurophysiological correlates. The first Nogo condition was used to test whether the generally altered tone processing affects the subsequent inhibitory processes in AP musicians. The second Nogo condition, on the other hand, might generate a Stroop-like effect: the two piano tones, although slightly different in tone frequency, belonged to the same pitch category and should, therefore, evoke the same pitch label in AP musicians. It has been shown before that AP musicians categorize mistuned tones in their nominal categories (e.g., a mistuned C will still be identified as C; Levitin and Rogers, 2005). If pitch labeling is difficult to suppress, AP musicians are expected to show some signs of increased inhibitory load due to the conflicting information, such as more commission errors and/or larger N2d/P3d amplitudes than non-AP musicians. Also, it has been suggested that AP musicians may have an aversion towards mistuned tones (Levitin and Rogers, 2005; Rogenmoser et al., 2020). This could increase the inhibitory load even further.

Finally, we also included a behavioral audio-visual Stroop task to confirm the presence of an incongruence effect as reported in previous studies in our sample of AP participants.

## Materials and Methods

### Participants

All 105 participants were recruited within a larger research project investigating the neural correlates of AP (Greber et al., 2018, 2020; Brauchli et al., 2019, 2020; Burkhard et al., 2019, 2020; Leipold et al., 2019a,b,c) and were professional musicians, music students, or highly-trained amateur musicians. In total, 54 musicians with AP and 51 musicians without AP participated in this study. The age of the participants ranged from 18 to 44 years. Participants were assigned to one of the two groups based on self-report in the initial online application form. This assignment was validated by an online pitch-labeling task (described below). If someone had self-identified as AP possessor but scored around or below the chance level of 8.3% in the pitch-labeling task, they were not invited to participate in the study. If someone had self-identified as non-AP possessor, which was confirmed again in the laboratory, and nonetheless achieved high scores in the pitch-labeling task, they were neither excluded from the study nor reassigned to the AP group.

Before being invited to the electroencephalography (EEG) recording, participants also filled out an online questionnaire assessing demographical information and musical experience. Based on these data, the two groups were matched for sex, age, handedness, age of onset of musical training, and cumulative hours of musical training over the lifespan.

None of the participants reported any neurological, severe psychiatric, or audiological disorders. We confirmed normal hearing thresholds in all participants using pure-tone audiometry (MAICO ST 20, MAICO Diagnostic, GmBh, Berlin) and validated self-reported handedness using a German translation of the Annett Handedness Questionnaire (Annett, 1970). Crystallized intelligence was evaluated with the Mehrfachwahl-Wortschatz-Intelligenztest (MWT-B; Lehrl, 2005), and fluid intelligence was evaluated with the Kurztest für Allgemeine Basisgrössen der Informationsverarbeitung (KAI: Lehrl and Fischer, 1992). Musical aptitude was estimated using the Advanced Measures of Music Audiation (AMMA; Gordon, 1989). The AMMA consists of 30 pairs of piano melodies. Participants are asked to decide whether the two melodies are identical, different in rhythmical patterns, or different in tonal patterns. The test results in a rhythmical score, a tonal score, and a total score (equals the sum of rhythmical and tonal score). Participant characteristics for the two groups are given in Table 1.

**Table 1:** Participant characteristics.

	Absolute Pitch Musicians (*n* = 54)	Non-Absolute Pitch Musicians (*n* = 51)
Sex
Female	27	24
Male	27	27
Age (years)	26.67 (5.49)	25.37 (4.49)
Handedness
Right-handed	47	46
Left-handed	4	4
Both-handed	3	1
Intelligence (MWT-B)^a^	27.69 (5.10)	29.10 (4.64)
Intelligence (KAI)^a^	123.41 (32.16)	132.19 (26.16)
Age of Onset of Musical Training (years)	5.93 (2.39)	6.49 (2.44)
Lifetime Cumulative Training (hours)^b^	1.66 (1.22)	1.35 (0.96)
Musical Aptitude (AMMA)^a^—total	66.11 (6.31)	63.35 (6.86)
Musical Aptitude (AMMA)^a^—total	32.33 (3.75)	30.45 (4.13)
Musical Aptitude (AMMA)^a^—rhythmical	33.78 (2.83)	32.90 (3.03)
Pitch-labeling Test (%)	76.41 (19.55)	24.04 (18.92)

*Continuous measures are given as mean (standard deviations in parentheses). MWT-B, Mehrfachwahl-Wortschatz-Intelligenztest; AMMA, Advanced Measures of Music Audiation. ^a^Raw scores. ^b^Units are given in 1 × 10^4^*.

The study was approved by the ethics committee of the canton of Zurich^1^ and was conducted in accordance with the Declaration of Helsinki. All participants provided written informed consent and received payment for their participation.

### Pitch-Labeling Task

As described above, participants completed an online pitch-labeling task at home before being invited to the laboratory. During the task (adapted from Oechslin et al., 2010), participants were instructed to identify both the pitch chroma (class, e.g., C) and the pitch height (octave, e.g., 4) of 108 pure tones. Tones ranged from C3 to B5 (tuning: A4 = 440 Hz) and had a duration of 500 ms. Immediately before and after each tone, 2,000 ms of Brownian noise were presented. In total, each tone was presented three times in a pseudorandomized order, ensuring that tones were not repeated in consecutive trials. Participants responded by selecting a label from a list of all possible labels (C3 to B5) within a maximal trial duration of 15,000 ms. Following the same scoring procedure as the other studies within the AP project (Greber et al., 2018, 2020; Brauchli et al., 2019, 2020; Burkhard et al., 2019, 2020; Leipold et al., 2019a,b,c), we quantified pitch-labeling ability as the percentage of correctly identified pitch classes without considering octave errors (Deutsch, 2013). We did not assign full or partial points to semitone errors. Accordingly, the chance level was at 8.3%.

### Auditory-Visual Stroop Task

An auditory-visual Stroop task (Stroop, 1935) was administered in the laboratory to assess the automaticity of pitch labeling (Allport et al., 1994; Itoh et al., 2005; Akiva-Kabiri and Henik, 2012; Schulze et al., 2013). This task has already been reported in another study for the same sample within the larger project on AP (Leipold et al., 2019b). During the task, auditory and visual stimuli were presented simultaneously. Both auditory and visual stimuli corresponded to C4, D4, E4, F4, and G4. The five auditory stimuli were pure tones with a duration of 500 ms (10 ms linear fade-in; 50 ms linear fade-out), created using Audacity (version 2.1.2)^2^. The visual stimuli consisted of the matching musical notations as quarter notes in treble clef. During the simultaneous presentation, the label of the tone and the name of the musical notation were either congruent or incongruent. Participants were asked to identify the visually presented musical notations as fast and accurately as possible by button press (keys labeled as C, D, E, F, or G) and to ignore the acoustically presented tones. If pitch labeling in AP musicians is automatic and difficult to suppress, AP musicians are expected to experience more cognitive interference in incongruent trials than non-AP musicians. This would be reflected by greater differences in response time between congruent and incongruent trials in AP musicians.

Response times were averaged separately for each participant and condition. Incorrect trials and response times that deviated by more than two standard deviations from the corresponding participant-and-condition-specific mean were excluded from the analysis. For each participant, we subtracted the mean response time of the congruent trials from the mean response time of the incongruent trials to quantify the Stroop effect. These differences in response times between congruent and incongruent trials were subjected to statistical group comparison.

### EEG Experiment: Auditory Go/Nogo Continuous Performance Task

During EEG recording, participants performed an auditory continuous performance task (ACPT) requiring Go/Nogo decisions. The auditory stimuli consisted of piano tones and environmental sounds. We used piano tones instead of pure tones for this task because the pitches of piano tones are usually easier to identify than those of piano tones (Miyazaki, 1989; Van Hedger and Nusbaum, 2018; Gruhn et al., 2019). Easy recognition of the pitch class was essential so that conflicting information regarding the sameness of the stimuli could potentially arise in the second Nogo condition.

Initially, five white-key piano tones (C4, D4, E4, F4, and G4) and 10 environmental sounds (e.g., water splashes, knocking on wood) were recorded. These auditory stimuli were then preprocessed using the Audacity software (Version 2.1.2)^2^. They were all shortened to 500 ms and normalized. A linear fade-in and a linear fade-out were applied to the first and last 100 ms respectively. Additional mistuned piano tones were generated by shifting the pitch of each of the originally recorded piano tones $\frac{1}{4}$ semitone (=25 cents) to sharp and to flat. In total, five in-tune piano tones, 10 mistuned piano tones, and 10 environmental sounds were used in the experiment.

The ACPT task consisted of 400 trials. Before starting the task, participants were asked to perform a few practice trials to check whether they had understood the task instruction. After 200 trials, participants were allowed to take a short break.

In each trial, two of the auditory stimuli were presented one after the other *via* Bose Companion 2 Series III external speakers (Bose Corporation, Framingham, MA, USA) at a sound pressure of approximately 75 dB using the ERPrec software (Version 2.0.x, Bee Medic GmbH, Germany). Trials lasted 3,800 ms with an interstimulus interval of 1,000 ms. The first stimulus was presented 300 ms after the start of the trial for a duration of 500 ms. After 500-ms presentation of the second stimulus, participants were given 1,500 ms to indicate a response. A black fixation cross on a white background was presented on the screen during the entire task.

**Figure 1:**
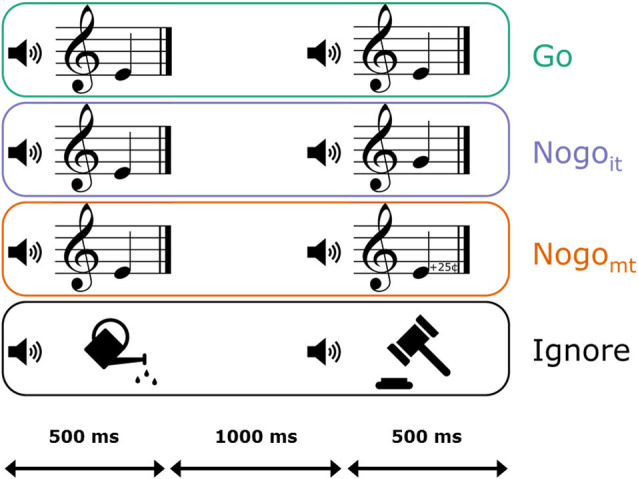
Graphical representation of the four trial categories in the auditory continuous performance task (ACPT). Participants were instructed to press a mouse button as fast and accurately as possible when a piano tone was followed by an identical piano tone (Go, depicted in green). When the second piano tone was a non-identical in-tune piano tone (Nogo_it_, depicted in violet) or a mistuned variant of the first piano tone (Nogo_mt_, depicted in orange; 25-cents deviation of the first stimulus), participants had to inhibit the prepared response. When the first stimulus was an environmental noise instead of a piano tone, no response had to be prepared (Ignore, depicted in black). Stimuli had a duration of 500 ms, and the interstimulus interval had a duration of 1,000 ms.

There were four different trial categories: Go trials, Nogo trials with in-tune tones (Nogo_it_), Nogo trials with mistuned tones (Nogo_mt_), and Ignore trials (compare Figure 1). All four trial categories were presented in randomized order and with equal probability (100 trials each). Participants were instructed to press the left mouse button with the right index finger as quickly and as accurately as possible whenever two identical piano tones were presented successively. The first stimulus was either an in-tune piano tone or an environmental sound. Thus, a piano tone as the first stimulus served as a cue for a potential button press, whereas an environmental sound indicated that no action was necessary (Ignore trial). In Go trials, the first piano tone was followed by an identical piano tone, thus requiring a button press. In Nogo_it_ trials, the second stimulus was also an in-tune piano tone but belonging to a different pitch class (e.g., E4 followed by G4). In Nogo_mt_ trials, the second stimulus was one of the slightly mistuned variants of the first stimulus (e.g., E4 followed by the 25-cents-sharp deviation of E4). In both Nogo_it_ and Nogo_mt_ trials, participants had to withhold pressing the button. In Nogo_mt_ trials, pitch labels of the two successive tones were identical, but pitch height was not. Thus, in the case of automatic pitch labeling, these trials contain conflicting information about the sameness of the two stimuli. If potential automatic labeling interferes with the task demands, AP musicians should demonstrate signs of a higher inhibitory load (i.e., larger N2d_mt_ or P3d_mt_ amplitudes, and/or higher error rates) compared to non-AP musicians in Nogo_mt_ trials.

Performance in the ACPT task was quantified as mean response time, number of omission errors, and number of commission errors. Response times were analyzed for correct Go trials and were measured as the time elapsed between the onset of the second stimulus and button press. Response times shorter than 200 ms and longer than 1,500 ms were not included in the average. Failures to respond in Go trials were counted as omission errors. Failures to inhibit a button press in Nogo_it_ and Nogo_mt_ trials were counted as commission errors. Trials in which a button press occurred between the first and the second stimulus were excluded from the behavioral analysis.

### EEG Recording and Preprocessing

For the EEG recording, participants were seated in an electrically shielded room and were instructed to fixate their gaze on a black cross on a white screen during the task. Before the experimental task, 3 min of eyes-open and 3 min of eyes-closed resting state were acquired. Continuous EEG was recorded from 31 scalp sites using an electrode cap (Comby EEG Cap, Pamel, Croatia), a Neuroamp^®^x39 amplifier (Bee Medic GmbH, Germany), and the ERPrec recording software (Version 2.0.x, Bee Medic GmbH, Germany). The silver/silver chloride electrodes were placed according to a subset of the 10/10 system (Fp1, Fpz, Fp2, F7, F3, Fz, F4, F8, FT7, FC3, FCz, FC4, FT8, T3, C3, Cz, C4, T4, TP7, CP3, CPz, CP4, TP8, T5, P3, Pz, P4, T6, O1, Oz, O2) and referenced to linked earlobes. Impedances of all electrodes were kept below 10 kΩ using an abrasive, electrically conductive gel (OneStep EEG-Gel, H + H Medizinprodukt GbR, Germany). The sampling rate was 500 Hz and no online filters were applied. After recording, data was converted to EDF+ using the xdf2eeg file converter implemented in the ERPrec software. During file conversion, a high-pass filter (Butterworth, 1st order) of 0.16 Hz and a fixed range scaling factor were applied to the EEG signal.

The converted data was subsequently preprocessed using the BrainVision Analyzer software package (Version 2.1, BrainProducts, Germany)^3^. First, the data was filtered with a bandpass filter (Butterworth, 8th order) of 1–30 Hz and a notch filter of 50 Hz. Next, eye movement artifacts were corrected using a restricted infomax independent component analysis (ICA; Jung et al., 2000). Noisy channels were excluded from the ICA and interpolated after ICA correction. Remaining artifacts were marked using an automatic raw data inspection with the following exclusion criteria: amplitude gradient >50 μV/ms, amplitude difference >100 μV within an interval of 200 ms, amplitude <−100 μV, amplitude >+100 μV, and activity <0.5 μV within an interval of 100 ms.

**Figure 2:**
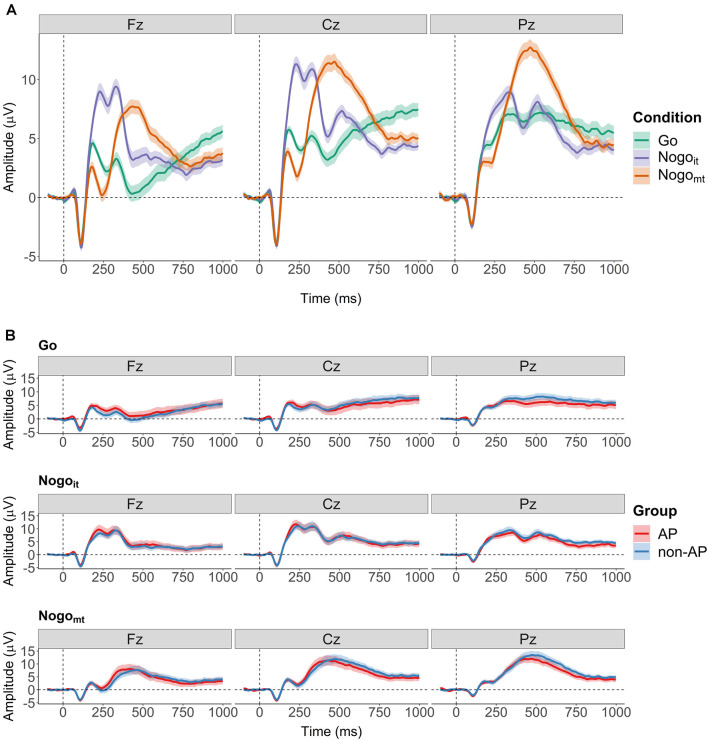
Grand averages of the event-relatedpotentials (ERPs) evoked by the second stimulus. **(A)** Grand averages over all participants for the three cued conditions (Go in green, Nogo_it_ in violet, and Nogo_mt_ in orange). Shaded areas depict the 95% within-subject confidence interval. **(B)** Grand averages computed separately for AP musicians (red) and non-AP musicians (blue). Shaded areas depict the 95% between-subject confidence interval.

Then, ERPs evoked by the second stimulus were computed separately for the three cued conditions (Go, Nogo_it_, and Nogo_mt_) and for each participant. The EEG signal was divided into artifact-free segments of 1,100 ms (−100 to +1,000 ms from the onset of the second stimulus), and baseline correction (−100 to 0 from the onset of the second stimulus) was applied. Only trials with a correct response (button press in Go; no button press in Nogo_it_ and Nogo_mt_) were included in the ERP averages. Grand and group averages of the ERPs at Fz, Cz, and Pz are shown in Figure 2. Supplementary Figure 1 shows the grand averages of the ERPs at all 31 electrodes. Supplementary Figures 2, 3, 4 show the ERPs at all 31 electrodes separately for the two groups.

**Figure 3:**
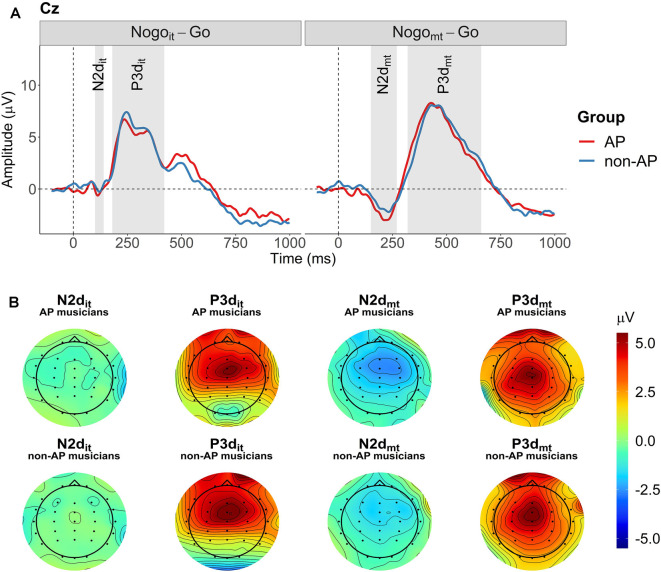
Grand averages and topographies of the difference waves (Nogo_it_ minus Go, Nogo_mt_ minus Go). **(A)** Grand averages of the two difference waves are shown separately for the two groups at electrode Cz (red: AP musicians, blue: non-AP musicians). Time windows used for the computation of mean amplitudes are indicated by the gray-shaded areas. **(B)** Topographies for the four ERP components of interest (N2d_it_, P3d_it_, N2d_mt_, and P3d_mt_) are shown separately for AP and non-AP musicians.

Two difference waves were computed by subtracting the participant-specific ERP evoked in the Go condition from the participant-specific ERPs evoked in the two inhibition conditions (Nogo_it_ minus Go and Nogo_mt_ minus Go). The N2 and P3 ERP components on the difference waves (N2d and P3d) were quantified as mean amplitudes at the three midline electrodes Fz, Cz, and Pz. Compared to peak amplitudes, mean amplitudes are more robust, less affected by latency variability between trials, and not biased by the noise level and the number of trials (Clayson et al., 2013; Luck, 2014). The definition of the time windows was based on the grand averages of the difference waves over all participants at electrode Cz (compare Figure 3). Because the onset and expansion of N2d and P3d differed between the two conditions, separate time windows were selected for Nogo_it_-Go and Nogo_mt_-Go. From now on, ERP components obtained from the difference wave between Nogo_it_ and Go will be referred to as N2d_it_ and P3d_it_. ERP components obtained from the difference wave between Nogo_mt_ and Go will be referred to as N2d_mt_ and P3d_mt_. Mean amplitudes were computed for N2d_it_ between 100 and 140 ms, for P3d_it_ between 180 and 420 ms, for N2d_mt_ between 150 and 270 ms, and for P3d_mt_ between 320 and 660 ms after stimulus onset. Time windows and topographies of the components are shown in Figures 3A,B, respectively. Visualizations of topographies and ERPs were created using functions from the R package EEGutils (Craddock, 2018). Supplementary Figure 5 shows the difference waves at all 31 electrodes averaged across all participants. Supplementary Figures 6, 7 show the difference waves at all 31 electrodes separately for the two groups.

Mean amplitudes and time series of the difference wave ERPs at electrodes Fz, Cz, and Pz were exported for statistical analyses.

### Statistical Analysis

All statistical analyses were performed using R (version 3.4.3^4^; R Core Team, 2017). The significance level was set to *α* = 0.05 for all statistical analyses unless stated otherwise. We compared the participant characteristics, the AMMA scores (rhythmical, tonal, and total), the Stroop effect, and the pitch-labeling score between the two groups using two-tailed Welch’s *t*-tests. Additionally, we computed Pearson correlations between the pitch-labeling scores and the Stroop effect across all participants as well as within each group.

Group differences in the performance measures of the ACPT (i.e., mean response times, omission errors, commission errors in Nogo_it_ trials, and commission errors in Nogo_mt_ trials) were also evaluated using Welch’s *t*-tests. Effect sizes for *t*-tests are given as Cohen’s *d* (Cohen, 1988).

To analyze group differences in the EEG data, we used two different approaches. First, we performed a traditional ERP-component analysis: we compared the N2d and P3d mean amplitudes between the two groups separately for the Nogo_it_-Go and the Nogo_mt_-Go condition. For each component (N2d_it_, P3d_it_, N2d_mt_, P3d_mt_), we computed a 2 × 3 ANOVA with between-subject factor Group (AP and non-AP) and within-subject factor Electrode Site (Fz, Cz, Pz) using the R package *ez* (version 4.4.0^5^; Lawrence, 2016). *P*-values and degrees of freedom were adjusted with the Greenhouse-Geisser correction for nonsphericity when appropriate. Effect sizes for the main effects and interactions are given as generalized eta-squared ($\eta_{\text{G}}^{2}$, Bakeman, 2005). To quantify the relative evidence of the alternative hypothesis (H1) and the null hypothesis (H0), we additionally report Bayes factors for the mean amplitudes. Bayes factors compare the (marginal) likelihood of the data between two hypotheses (i.e., H1 and H0). Contrary to frequentist statistics, this allows for conclusions about the evidence in support of H0 (Dienes, 2011, 2014). The likelihood ratio expressed by a Bayes factor can be interpreted as follows: A BF_10_ of 5 (or the inverse $\frac{1}{{\text{BF}}_{10}}$ = BF_01_ of 0.2) indicates that the observed data is five times more likely under H1 than under H0. To make the interpretation more straightforward for the reader, we report BF_10_ when the relative evidence is in favor of H1, and BF_01_ when the relative evidence is in favor of H0.

We computed the Bayes factors using the R package *BayesFactor* (version 0.9.12-4.2^6^; Morey et al., 2018). We used the default settings implemented in the *BayesFactor* package for the number of iterations (*n* = 10,000) and for the prior scale parameter (*r* = 0.707 for Bayesian *t*-tests; *r* = 0.5 for Bayesian ANOVAs). To assess the two main effects of the Bayesian ANOVAs (i.e., group and electrode), the model with one factor (e.g., group + subject) was compared to the model with both factors (e.g., group + electrode + subject). For the interaction effect, the full model (group + electrode + group * electrode + subject) was compared to the model without the interaction effect (group + electrode + subject).

Second, we adopted a more data-driven approach to analyze the difference waves. Using cluster-based permutation tests implemented in the R package *permuco* (version 1.0.2^7^ ; Frossard and Renaud, 2019), we performed a 2 × 2 ANOVA with between-subject factor Group (AP and non-AP) and within-subject factor Condition (Nogo_it_-Go and Nogo_mt_-Go) at each time point after stimulus onset (0 to 1,000 ms). This analysis was conducted separately for each of the three electrodes (Fz, Cz, and Pz). To control for multiple comparisons over time-points, threshold-free cluster enhancement (TFCE: Smith and Nichols, 2009; Mensen and Khatami, 2013) was combined with non-parametric maximum permutation statistics. The TFCE procedure incorporates neighborhood information (i.e., time points close to each other tend to correlate) and does not require an arbitrary cluster-forming threshold. The same procedure was repeated 5,000 times using randomly permuted datasets of the original dataset. From each permutation step, the maximal TFCE score was obtained to form an empirical null distribution, to which the TFCE scores from the original datasets were compared.

## Results

### Demographic and Behavioral Data

**Figure 4:**
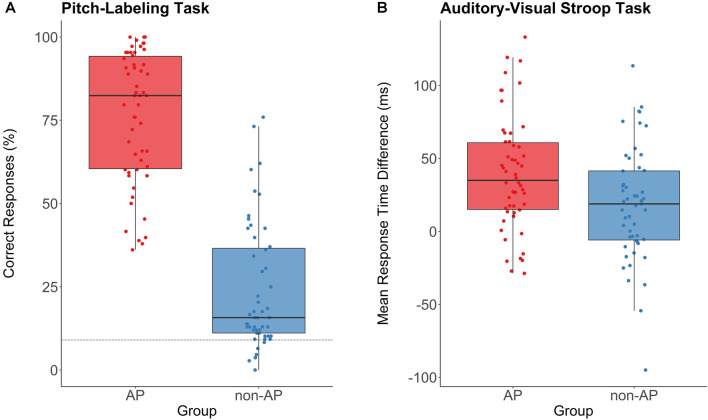
Performance in the pitch-labeling task and the auditory-visual Stroop task for AP musicians (*n* = 54, depicted in red) and non-AP musicians (*n* = 51, depicted in blue). **(A)** AP musicians showed a substantially better pitch-labeling ability. The dashed line represents the chance level of 8.3%. **(B)** The incongruence effect (response time difference between congruent and incongruent trials) in the Stroop task was more pronounced in AP musicians than in non-AP musicians. This indicates that the pitch labeling was difficult to suppress for our sample of AP musicians.

The two groups were comparable in age (*t*_(100.97)_ = 1.33, *p* = 0.19, *d* = 0.26), crystallized intelligence (MWT-B: *t*_(102.86)_ = −1.49, *p* = 0.14, *d* = 0.29), fluid intelligence (KAI: *t*_(100.82)_ = −1.54, *p* = 0.13, *d* = 0.30), age of onset of musical training (*t*_(102.42)_ = −1.20, *p* = 0.23, *d* = 0.23), and cumulative musical training hours over the lifespan (*t*_(99.71)_ = 1.43, *p* = 0.16, *d* = 0.28). AP musicians scored slightly higher in the AMMA total score (*t*_(100.99)_ = 2.14, *p* = 0.035, *d* = 0.42). Analyses of the subtests revealed that this effect was driven by higher AMMA tonal scores in AP musicians (*t*_(100.61)_ = 2.44, *p* = 0.016, *d* = 0.48). In the AMMA rhythmical score, AP and non-AP musicians were comparable (*t*_(101.38)_ = 1.53, *p* = 0.13, *d* = 0.30). In the pitch-labeling task, AP musicians performed considerably better than non-AP musicians (*t*_(102.93)_ = 13.95, *p* < 0.001, *d* = 2.72; see Figure 4A). In the auditory-visual Stroop task, AP musicians showed a larger incongruence effect than non-AP musicians (*t*_(102.65)_ = 2.78, *p* = 0.007, *d* = 0.54; see Figure 4B), indicating difficulties to suppress pitch labeling in this task. Across the whole sample, pitch-labeling scores were positively correlated with the size of the incongruence effect in the auditory-visual Stroop task (*r* = 0.24, *p* = 0.015). Within the groups, there was no evidence for a relationship between pitch-labeling scores and the size of the Stroop effect (AP: *r* = −0.12, *p* = 0.37; RP: *r* = 0.22, *p* = 0.12).

### ACPT Performance Data

**Figure 5:**
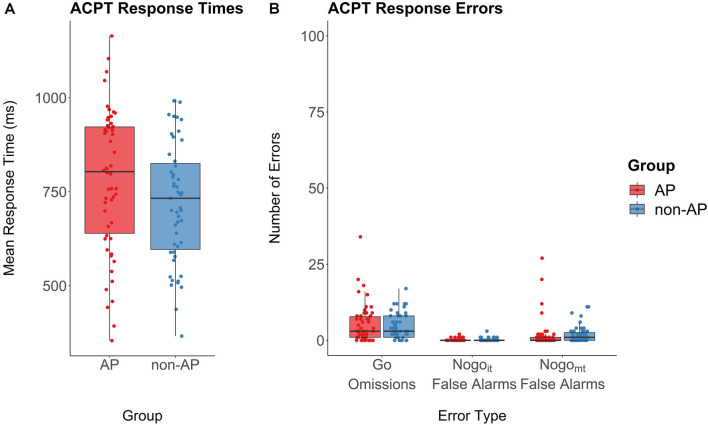
Performance in the ACPT. **(A)** Response times in Go trials revealed no evidence for a group difference between AP musicians (red) and non-AP musicians (blue). **(B)** Response error rates for omissions (no button press in Go trials) and false alarms (failure to inhibit button press in Nogo_it_ and Nogo_mt_ trials). There was no evidence for a group difference with regard to the three error types.

Musicians with AP and musicians without AP showed comparable error rates for omission errors (*t*_(92.40)_ = 0.70, *p* = 0.48, *d* = 0.14), commission errors in Nogo_it_ trials (mean AP musicians = 0.19, SD AP musicians = 0.44, mean non-AP musicians = 0.22, SD non-AP musicians = 0.54; *t*_(96.23)_ = −0.32, *p* = 0.75, *d* = 0.06), and commission errors in Nogo_mt_ trials (mean AP musicians = 1.76, SD AP musicians = 4.83, mean non-AP musicians = 1.96, SD non-AP musicians = 2.69; *t*_(83.95)_ = −0.27, *p* = 0.79, *d* = 0.05). Response times in Go trials were on average slightly longer in AP musicians (mean response time = 781.37 ms, SD = 188.27 ms) than in non-AP musicians (mean response time = 719.94 ms, SD = 159.40 ms), but the difference was not statistically significant (*t*_(101.81)_ = −1.81, *p* = 0.073, *d* = 0.35). Performance measures are shown in Figures 5A,B.

### EEG Data: N2d and P3d Mean Amplitudes

**Figure 6:**
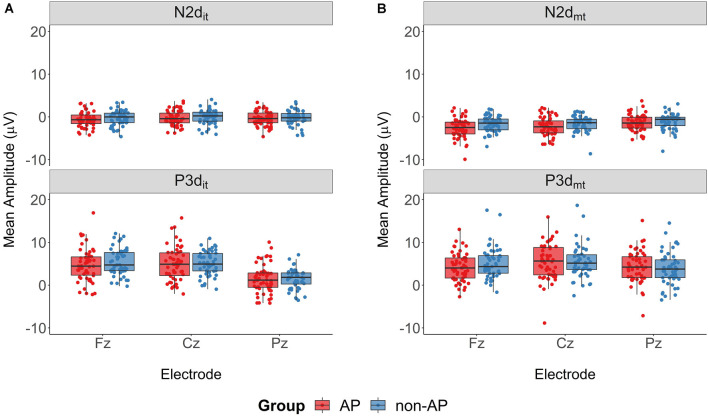
Mean amplitudes of the ERP components. **(A)** ERP components of the Nogo_it_-Go difference wave. AP musicians (depicted in red) and non-AP musicians (depicted in blue) had comparable N2d_it_ and P3d_it_ mean amplitudes. **(B)** ERP components of the Nogo_mt_-Go difference wave. The 2 × 2 ANOVA revealed a Group × Electrode interaction for the P3d_mt_ component. *Post hoc* analyses showed no evidence for a difference between AP and non-AP musicians at any electrode site.

Mean amplitudes of the ERP components are shown in Figure 6. The two-way ANOVA of the N2d_it_ amplitudes did not reveal a main effect of Group (*F*_(1.103)_ = 0.82, *p* = 0.37, $\eta_{\text{G}}^{2}$ = 0.006, BF_01_ = 2.32), a main effect of Electrode (*F*_(1.33,137.16)_ = 2.16, *p* = 0.14, $\eta_{\text{G}}^{2}$ = 0.004, BF_01_ = 4.03), or an interaction effect (*F*_(1.33,137.16)_ = 0.12, *p* = 0.80, $\eta_{\text{G}}^{2}$ < 0.001, BF_01_ = 14.10). The analysis of the P3d_it_ amplitudes also revealed no main effect of Group (*F*_(1.103)_ = 0.24, *p* = 0.62, $\eta_{\text{G}}^{2}$ = 0.002, BF_01_ = 2.68) and no Group × Electrode interaction (*F*_(1.41,145.26)_ = 0.77, *p* = 0.42, $\eta_{\text{G}}^{2}$ = 0.001, BF_01_ = 8.94), but did reveal a main effect of Electrode (*F*_(1.41,145.26)_ = 145.92, *p* < 0.001, $\eta_{\text{G}}^{2}$ = 0.21, BF_10_ = 1.04 * 10^37^). According to pairwise comparisons, P3d_it_ amplitudes were smaller at Pz (mean = 1.49 μV, SD = 2.84 μV) than at Fz (mean = 5.06 μV, SD = 3.49 μV, *t*_(104)_ = 11.60, *p* < 0.001, *d* = 1.13, BF_10_ = 2.62 * 10^17^) and at Cz (mean = 5.03 μV, SD = 3.43 μV, *t*_(104)_ = 18.89, *p* < 0.001, *d* = 1.84, BF_10_ = 1.23 * 10^32^).

The two-way ANOVA of the N2d_mt_ amplitudes similarly revealed a main effect of Electrode (*F*_(1.32,135.71)_ = 27.50, *p* < 0.001, $\eta_{\text{G}}^{2}$ = 0.035, BF_10_ = 2.25 * 10^8^). Again pairwise comparisons showed that amplitudes were less pronounced at electrode Pz (mean = −1.25 μV, SD = 1.93 μV) than at electrode Fz (mean = −2.12 μV, SD = 2.12 μV, *t*_(104)_ = −5.25, *p* < 0.001, *d* = 0.51, BF_10_ = 1.62 * 10^4^) and electrode Cz (mean = −1.96 μV, SD = 2.02 μV, *t*_(104)_ = −7.05, *p* < 0.001, *d* = 0.69, BF_10_ = 4.62 * 10^7^). We found no main effect of Group (*F*_(1.103)_ = 2.87, *p* = 0.093, $\eta_{\text{G}}^{2}$ = 0.024, BF_01_ = 1.01) nor an interaction effect (*F*_(1.32,135.71)_ = 2.93, *p* = 0.078, $\eta_{\text{G}}^{2}$ = 0.004, BF_01_ = 1.20) for the N2d_mt_ amplitudes.

The evaluation of the P3d_mt_ amplitudes did not reveal a main effect of Group (*F*_(1.103)_ = 0.05, *p* = 0.82, $\eta_{\text{G}}^{2}$ < 0.001, BF_01_ = 2.52), but did reveal a main effect of Electrode (*F*_(1.50,154.31)_ = 21.89, *p* < 0.001, $\eta_{\text{G}}^{2}$ = 0.022, BF_10_ = 1.89 * 10^6^) and a Group × Electrode interaction (*F*_(1.50,154.31)_ = 6.22, *p* = 0.006, $\eta_{\text{G}}^{2}$ = 0.006, BF_10_ = 12.69). The *post hoc* *t*-tests comparing the P3d_mt_ amplitudes between the two Groups at each electrode provided no evidence for a difference between AP and non-AP musicians at any of the three electrodes (Fz: *t*_(100)_ = −1.40, *p* = 0.16, *d* = 0.27, BF_01_ = 2.01; Cz: *t*_(102.9)_ = 0.006, *p* = 0.995, *d* = 0.001, BF_01_ = 4.85; Pz: *t*_(101.9)_ = 0.65, *p* = 0.52, *d* = 0.13, BF_01_ = 4.01).

### EEG Data: Cluster-Based Permutation Test

**Figure 7:**
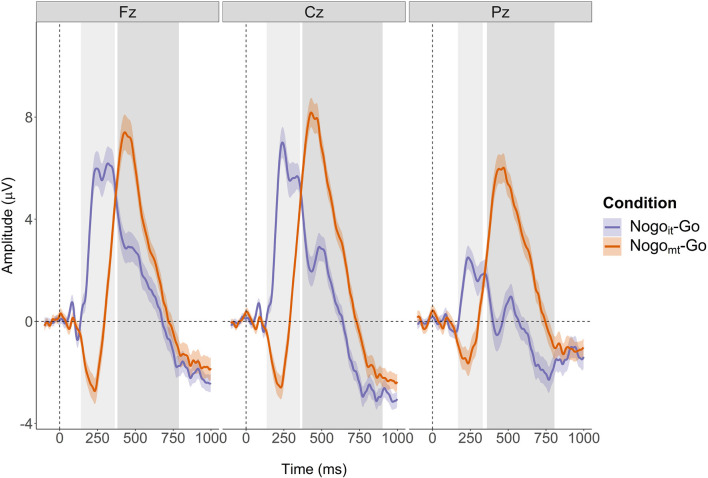
Cluster-based permutation tests revealed a main effect of Condition at each of the three electrodes (Fz, Cz, and Pz). Clusters are indicated by grey-shaded areas. The N2d and P3d of the Nogo_mt_-Go difference wave (depicted in orange) appeared later after stimulus onset than the corresponding ERP components of the Nogo_it_-Go difference wave (depicted in violet). The Nogo_it_-Go difference wave showed a considerably smaller N2d amplitude at all electrodes as well as a smaller P3d amplitude at electrode Pz than the Nogo_mt_-Go difference wave.

The non-parametric cluster-based permutation tests indicated an effect of condition at all three analyzed electrode sites (*p* = 0.0002). This corresponded to two clusters in the analyzed time window at each electrode site. At electrode Fz, the first cluster extended from 140 to 364 ms, and the second cluster extended from 382 to 788 ms. At electrode Cz, the effect was driven by a cluster between 136 and 356 ms, and a cluster between 372 and 902 ms. At electrode Pz, the corresponding clusters extended from 168 to 332 ms and from 360 to 806 ms. The clusters are shown in Figure 7. Descriptively, N2d and P3d amplitudes were time-shifted between the two conditions, giving rise to the two detected clusters. Additionally, N2d amplitudes were substantially smaller in the Nogo_it_-Go condition compared to the Nogo_mt_-Go condition.

**Figure 8:**
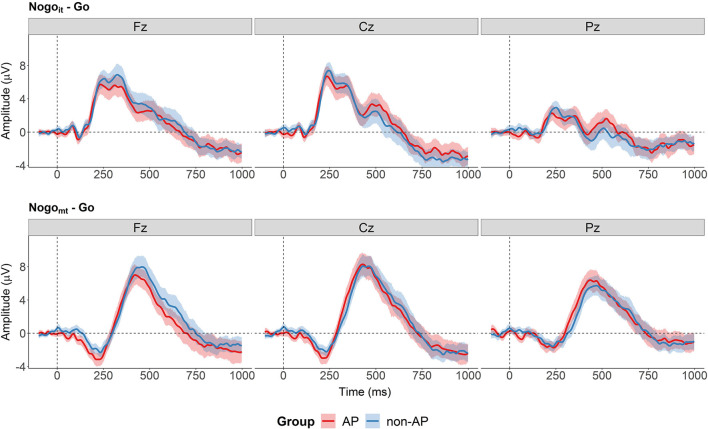
Difference waves separately for the two groups at the three electrodes (Fz, Cz, and Pz). Cluster-based permutations tests provided no evidence for a group difference between AP and non-AP musicians at any of the electrodes of the difference waves. The shaded areas represent the 95% between-subject confidence interval.

Regarding group differences, the cluster-based permutation tests revealed no evidence for an effect of Group nor an interaction between Group and Condition at any of the three electrode sites. All clusters found for these effects had *p*-values above 0.15. The difference waves at electrodes Fz, Cz, and Pz are shown in Figure 8 separately for the two conditions and the two groups.

## Discussion

In the present study, we investigated whether the postulated highly automatic pitch labeling in AP affects subsequent inhibitory processes. We used a cued auditory Go/Nogo task requiring same/different judgments for pairs of consecutively presented piano tones. In Go trials, the two piano tones were identical. In Nogo_it_ trials, the second piano tone was always in-tune and differed at least one semitone from the first piano tone. In Nogo_mt_ trials, the second tone was a $\frac{1}{4}$-semitone mistuned variant of the first piano tone. While the Nogo_it_ condition tested if auditory-related inhibitory processes are generally altered in AP musicians, the Nogo_mt_ condition tested more specifically the suppressibility of pitch labeling by introducing contradictory information about the sameness of the stimuli (same pitch class, different tone frequency). We analyzed both behavioral and electrophysiological measures to evaluate a potential change in inhibitory load in AP musicians. For the electrophysiological measures, we adopted two analysis approaches: First, we conducted a traditional ERP analysis using mean amplitudes for the N2d and P3d components. Second, we performed a cluster-based permutation analysis to test the complete ERP segment. Our data did not provide evidence for a group difference in commission errors, N2d amplitudes, P3d amplitudes, or overall difference wave ERPs for either of the two Nogo conditions. There was also no evidence for a group difference in response times and omission errors in Go trials.

Previous neurophysiological studies have repeatedly reported group differences between AP and non-AP musicians during attentive listening without labeling instruction (Zatorre et al., 1998; Ohnishi et al., 2001; Wu et al., 2008; Wengenroth et al., 2014; Burkhard et al., 2019; Leipold et al., 2019a). In the current study, we tested whether these AP-specific alterations in neurophysiological activity modify the need for inhibition. The behavioral and electrophysiological results obtained from the Nogo_it_ trials do not support this hypothesis: The inhibitory processes in an auditory Go/Nogo task do not seem to have been influenced by absolute pitch. Whether or not the differential tone processing affects subsequent cognitive processes and the associated neurophysiological measures, may strongly depend on the specific task. Such a dependence of AP-specific effects on situational factors has previously been demonstrated with regard to the influential finding of reduced P3b amplitudes in AP musicians in active auditory oddball tasks (Klein et al., 1984). Since the P3b is thought to reflect working memory processes (for a review, see Kok, 2001; Polich, 2007), it was inferred from the original finding of smaller P3b amplitudes that AP musicians may not need to update their working memory during the task because they can access permanent pitch templates. Some of the subsequent studies replicated the effect (Hantz et al., 1992; Wayman et al., 1992; Crummer et al., 1994) while others did not (Hantz et al., 1995; Hirose et al., 2002). Bischoff Renninger et al. (2003) integrated these heterogeneous findings by demonstrating that the AP musicians employed different listening strategies (i.e., absolute pitch or relative pitch) depending on task difficulty and task instruction. Active oddball tasks are structured similarly to Go/Nogo tasks, but target tones appear only infrequently (Kropotov, 2009). The instruction to focus on tone-frequency changes in the current study might have encouraged a relative-pitch rather than an absolute-pitch listening strategy, preventing us from observing AP-specific effects.

Furthermore, the listening instruction might not only influence later task-related cognitive processes in AP musicians but also the differential tone processing *per se*. As described above, group differences between AP and non-AP musicians in neurophysiological activity have been repeatedly observed during attentive listening. However, in several studies, the mismatch negativity, an ERP component evoked during passive listening in passive oddball tasks, did not differ between AP and non-AP musicians (Tervaniemi et al., 1993; Rogenmoser et al., 2015; Greber et al., 2018). Thus, the focus of attention could play a role in whether and to what extent subprocesses of pitch labeling and associated neurophysiological activations are automatically triggered by acoustic stimuli. In the current study, participants did have to pay close attention to the presented tones but were instructed to concentrate on one specific dimension of the stimuli (i.e., the sameness of tone frequency), which was independent of the pitch class. In contrast to findings during attentive listening without an additional task (Burkhard et al., 2019), the visual inspection of the group-averaged ERPs (Figure 2B) shows comparable N1 and P2 amplitudes in response to the Go stimulus for AP and non-AP musicians. This could indicate that the AP-specific neurophysiological activity thought to be automatically induced by simply listening to tones was not present in our task, and, consequently, no additional inhibitory efforts would have been required.

Finally, it is also possible that the measures used in the current study were simply not sensitive enough to capture subtle group differences in inhibition. For instance, a ceiling effect occurred for both AP and non-AP musicians’ task performance, which was most evident in Nogo_it_ trials. Since the musicians had no difficulty solving the task, possibly the small increases in inhibitory loads did not show up in the error rates.

Compared to the Nogo_it_ condition, the Nogo_mt_ condition additionally evaluated whether the AP musicians were able to suppress conflicting pitch-labeling information. The assumption that pitch-labeling information is difficult to suppress stems mainly from auditory Stroop tasks (Miyazaki, 2004; Itoh et al., 2005; Hsieh and Saberi, 2008; Akiva-Kabiri and Henik, 2012; Schulze et al., 2013). Usually, AP musicians are asked to name a sung syllable or to label a visually presented note while ignoring the pitch of the sung syllable or a simultaneously presented musical tone. AP musicians reliably show an increased interference effect of incongruent stimuli or incongruent stimulus dimensions compared to non-AP musicians. Consistent with the literature, our behavioral auditory-visual Stroop task evoked a greater incongruence effect in AP musicians than in non-AP musicians. This suggests that the auditory-visual Stroop task did activate pitch-labeling processes in our sample of AP musicians, which then interfered with the labeling of the visual notes.

In Nogo_mt_ trials, tone frequency and pitch class of the second stimulus also provided contradictory information in a Stroop-like manner. Hence, we expected that—analogous to the Stroop task—AP musicians would process both the task-relevant (i.e., tone frequency) and the task-irrelevant stimulus dimension (i.e., pitch-class) due to the automaticity of pitch labeling, resulting in an increased inhibitory load compared to non-AP musicians. Instead, we found no evidence for a group difference in behavioral or electrophysiological measures for Nogo_mt_. These results suggest that AP musicians can successfully control irrelevant pitch-label information in the context of a Go/Nogo task with same/different judgments. Given the results from the Stroop task, it appears that the task context and the corresponding task demands might critically influence whether conflicting pitch-labeling information hinders performance. Contrary to incongruent trials in the Stroop task, the pitch label (e.g., “C”) of the second tone—considered in isolation—had no semantic overlap with the required response (i.e., “different”) in the Nogo_mt_ trials. Rather the information extracted from the pitch labels of both tones (e.g., C followed by C equals the same pitch label; “same”) did not match the information of the tone frequency comparison (i.e., different tone frequencies; “different”). Contradictory pitch-labeling information might predominantly impair performance when the task itself specifically requires a response of the same semantic category (i.e., a musical label as in naming a visual note or a sung tone label). A recent study investigated the strength of association between pitch information and verbal labels in musicians using a Stroop paradigm (Sharma et al., 2019). The study included three different Stroop tasks that required high pitch/low pitch judgments of sung syllables tuned to either 261.3 Hz (C4; low) or 392 Hz (G4; high). The sung syllables could be congruent or incongruent with the pitch height in terms of English words (/low/ and /high/), English solemnizations (/do/ and /so/), or key notations (/see/ and /jee/). The incongruence effect on response times was attenuated for solemnizations compared to English words in both AP and non-AP musicians. For key notations, there was no evidence for an incongruence effect on response times. It appears that the verbal labels were semantically not as strongly mapped to the high/low response. Most interestingly, this was even the case for AP musicians. Although the sung label (as keycode or solemnization) was semantically incongruent with the pitch-labeling information, they showed comparable incongruence effects on response times as non-AP musicians. Linguistic information conflicting with pitch-labeling information did not further impair the task performance of AP musicians for high/low judgments. It should be noted that the AP musicians but not the non-AP musicians did show a significant incongruence effect on ERP measures in the keycode task. However, the absence of evidence for an incongruence effect in non-AP musicians is not sufficient to conclude that there is a group difference without a direct statistical group comparison of the effect. Taken together, AP musicians may be able to ignore task-irrelevant conflicts with pitch-labeling information depending on the specific task and context. Considering that automatic processes are often described as obligatory, stimulus-driven, and requiring little to no attention (Palmeri et al., 2004; Palmeri, 2006), the findings of the current study may indicate that pitch labeling in AP is less automatic than previously assumed.

It is, however, important to note that the evidence in favor of H0 as indicated by the Bayes factors was only anecdotal or inconclusive (BF_01_ < 3). To get more conclusive evidence within the Bayesian framework (i.e., that there is no difference between the ERPs of the two groups, or that there is a group difference), an even larger sample would be needed. Unfortunately, due to the rarity of AP as well as the time-consuming and resource-intensive data acquisition in neuroscience, it is challenging for a single research group to recruit large numbers of AP musicians. For future research, collaborations between multiple research groups might be helpful in this regard.

While there was no evidence for a group difference, the cluster-based permutation analysis did reveal an effect of condition for the ERP difference waves. Visual inspection of the two corresponding clusters (compare Figure 7) shows that the N2d of the Nogo_it_-Go ERP was vanishingly small at all electrodes analyzed (Fz, Cz, and Pz) whereas the N2d of the Nogo_mt_-Go ERP was more pronounced and prolonged. Also, the P3d was latency-shifted and slightly larger for the Nogo_mt_-Go ERP compared to the Nogo_it_-Go ERP.

Small or even absent N2 effects as in Nogo_it_ have been repeatedly observed in auditory Go/Nogo tasks (Schröger, 1993; Falkenstein et al., 1995, 1999, 2002; Kiefer et al., 1998). Initially, this phenomenon was attributed to the stimulus modality, as visual stimuli seemed to consistently elicit larger N2 effects (e.g., Falkenstein et al., 1995, 1999, 2002). However, later studies could demonstrate that the relative N2 amplitude may depend more on the perceptual overlap between target and non-target stimuli than on the modality (Nieuwenhuis et al., 2004; Azizian et al., 2006; Smith and Douglas, 2011). Non-target stimuli that are more similar to the target stimulus may generate a stronger tendency to (erroneously) respond, and, thus, require greater inhibition efforts (Azizian et al., 2006). Differences in perceptual similarity could explain the N2d condition difference found in the present study. In Nogo_mt_ trials, the target and the non-target stimuli were much more similar ($\frac{1}{4}$-semitone difference) than in Nogo_it_ trials (difference of at least one semitone). This was paralleled by an increase in N2d amplitude. The more pronounced and prolonged N2d for Nogo_mt_ then probably shifted the latency of the P3d. The P3d itself likewise showed a larger amplitude for Nogo_mt_ than for Nogo_it_, mainly noticeable at the parietal electrode Pz. Hence, the amplitude of the P3d might have also been sensitive to the degree of perceptual overlap. An increase in both amplitude and latency of Nogo-P3 due to higher stimulus similarity has been previously reported for visual stimuli (Azizian et al., 2006). A second study, on the other hand, found comparable P3 effects for similar and dissimilar stimuli in the auditory and visual domain (Smith and Douglas, 2011). However, even the similar acoustic stimuli there differed by 165 cents (1,000 Hz/1,100 Hz; a difference of about one and a half semitone) compared to 25 cents in the current study. Thus, the P3 effect might only be affected by a very high perceptual overlap.

Even though the condition effect appears to be consistent with previous findings on the perceptual similarity between target and non-target stimuli, we cannot exclude the possibility that the ERPs were additionally modulated by the tuning or mistuning of the tones. In our Go/Nogo task, the first piano tone was always in-tune in Go, Nogo_it_, and Nogo_mt_ trials because the intonation context can influence the pitch classification in AP musicians (Van Hedger et al., 2018). By constantly providing in-tune tones, we hoped to ensure that the AP musicians’ internal intonation matched the intonation of the tones. This, combined with the frequency spacing applied, resulted in the second tones being mistuned in all Nogo_mt_ trials and being in-tune in all Nogo_it_ trials. Therefore, we are not able to distinguish the contributions of the tuning of the second stimulus (in-tune vs. mistuned) from the contributions of the frequency distance between the first and second stimulus (≥1 semitone in Nogo_it_ vs. $\frac{1}{4}$ semitone in Nogo_mt_) to the condition difference. To disentangle these two effects, future studies could use mistuned tones with a greater frequency distance to the first stimulus (e.g., D4 followed by a sharp-mistuned F4). This would also allow to include non-musicians in the sample to evaluate the influence of musical experience, which unfortunately was not feasible with the current task paradigm. During pilot-testing, participants without musical training were not able to discriminate the $\frac{1}{4}$-semitone frequency changes in Nogo_mt_ trials. The small number of correct trials resulted in too few EEG segments (i.e., between one and six out of 100 Nogo_mt_ trials) to compute reliable ERPs. It is well established that non-musicians have higher discrimination thresholds than musicians (Spiegel and Watson, 1984; Micheyl et al., 2006). We, nevertheless, have deliberately chosen the $\frac{1}{4}$-semitone difference so that the second tone would still be recognized by AP musicians as belonging to the same pitch category as the first tone. Had we chosen a larger frequency difference (e.g., $\frac{1}{2}$ semitone) to the first tone (e.g., E4), the second tone might have been assigned to a different pitch category (e.g., E4♯ or E4♭).

Further limitations of our study concern the pitch-labeling task. As can be seen in Figure 4A, the pitch-labeling scores overlapped between the two groups, with some self-identified AP musicians performing worse than some self-identified non-AP musicians. This overlap may be attributed to three features of our pitch-labeling task. First, because participants had to choose one out of 36 possible response options, each trial could last up to 15 s. This relatively long response window was shown to be necessary during pilot tests. Unfortunately, this may have allowed some of the non-AP musicians to use their relative-pitch ability to solve the task. One possibility to better distinguish AP and non-AP musicians based on pitch-labeling performance would be to include both response accuracy and latency information in a combined score (as suggested by Bermudez and Zatorre, 2009). The reconstruction of pitch labels based on a relative-pitch strategy is expected to take more time than genuine AP (see also Miyazaki, 1990). In the current study, the online implementation in an unstandardized setting at home (e.g., some participants used a computer mouse, some a touch screen, and others the trackpad of their laptop to submit the responses) in combination with the 36-item multiple-choice format did not allow to collect meaningful response time data. Future studies could reduce the item list by only asking for the pitch chroma irrespective of the octave. For accurate response time measures, the response options could then be arranged in a circular shape with equal distance to the starting point of the cursor (e.g., as done in Sharma et al., 2019). Another possibility for a better distinction between AP and non-AP musicians would be to prevent non-AP musicians from accessing relative-pitch cues. Wengenroth et al. (2014) proposed inserting non-harmonic and distorted interference stimuli between the tones for this purpose. For AP musicians, unpublished data from our lab (*n* = 39) suggests a strong correlation (*r* = 0.7) between our online pitch-labeling task and the original paper-pencil test of our group (Oechslin et al., 2010), which had shorter interstimulus intervals (4 s) and was conducted in a controlled setting. Thus, the longer interstimulus interval in the online implementation probably affects non-AP musicians more strongly than AP-musicians.

A second feature of the pitch-labeling task that might have affected the score distribution is the use of pure tone stimuli. Pure tones do not give an advantage to any specific group of instrumentalists based on their familiarity with a timbre (see Takeuchi and Hulse, 1993). However, pitch identification is generally more challenging for pure tones than for instrumental sounds (Miyazaki, 1989; Gruhn et al., 2019). In a study by Van Hedger and Nusbaum (2018), self-reported AP possessors achieved an accuracy between 75% and 100% (mean: 95.4%) for a mixture of piano and guitar tones, but only an accuracy between 25% and 100% (mean: 56.4%) for pure tones. In our sample of AP musicians, the accuracy for pure tones was even slightly higher (range: 36.1%–100%, mean: 76.4%). Therefore, it is very well possible that our AP musicians would similarly have shown higher accuracy rates for instrumental sounds. Future studies might want to consider including both pure and instrumental tones to get a better estimate of pitch-labeling ability.

Third, the tones in the pitch-labeling test were tuned to a standard reference of A4 = 440 Hz. This might have disadvantaged AP musicians who often play music tuned to a different reference tone (e.g., baroque tuning). Studies that categorize AP and non-AP musicians based on pitch-labeling performance could incorporate information about the musicians’ primary reference tone in the scoring procedure.

Within our AP project, the pitch-labeling task was designed as an additional validation tool only. As set out above, it is not optimally suited to distinguish AP musicians from non-AP musicians by itself. Most importantly, using self-report, we did not have to apply an arbitrary cutoff for group construction and did not risk erroneously assigning well-performing non-AP musicians to the AP group.

## Conclusion

The current study provided no evidence for an effect of AP on behavioral or neurophysiological measures of inhibition in a cued auditory Go/Nogo task. The results from the Nogo_mt_ condition further suggest that AP musicians can suppress pitch-labeling information depending on the task demands. Given the results from the bimodal Stroop task, it remains unclear under which circumstances subprocesses of pitch labeling are activated and to what extent these processes can be considered automatic. While the ERPs were not modulated by AP, there was a condition difference between the two Nogo conditions which probably reflects a modulation by the perceptual similarity between target and non-target stimuli.

## Data Availability Statement

The datasets presented in this study can be found in online repositories. The names of the repository/repositories and accession number(s) can be found below: Open Science Framework: https://osf.io/f5nkx/.

## Ethics Statement

The studies involving human participants were reviewed and approved by Kantonale Ethikkommission Zürich. The patients/participants provided their written informed consent to participate in this study.

## Author Contributions

MG and LJ designed research and wrote the article. MG performed research and analyzed data. All authors contributed to the article and approved the submitted version.

## Conflict of Interest

The authors declare that the research was conducted in the absence of any commercial or financial relationships that could be construed as a potential conflict of interest.

## Footnotes

^1^http://www.kek.zh.ch

^2^http://www.audacityteam.org/

^3^https://www.brainproducts.com/

^4^https://www.r-project.org

^5^https://cran.r-project.org/web/packages/ez/index.html

^6^https://cran.r-project.org/web/packages/BayesFactor/index.html

^7^https://cran.r-project.org/web/packages/permuco/index.html
