# Supplementary material for: Suppression of Pitch Labeling: No Evidence for an Impact of Absolute Pitch on Behavioral and Neurophysiological Measures of Cognitive Inhibition in an Auditory Go/Nogo Task
Source: Front Hum Neurosci. 2020 Nov 12;14:585505. doi: 10.3389/fnhum.2020.585505 (PMC7688746; doi:10.3389/fnhum.2020.585505)
Supplement: Supplementary file 1 [file Data_Sheet_1.PDF]

Supplementary Material

Suppression of Pitch Labeling: No Evidence for an Impact of Absolute Pitch on Behavioral and Neurophysiological Measures of Cognitive Inhibition in an Auditory Go/Nogo Task

Marielle Greber Lutz Jäncke

28 September 2020

Condition comparison: Go, Nogo$_{it}$, Nogo$_{mt}$

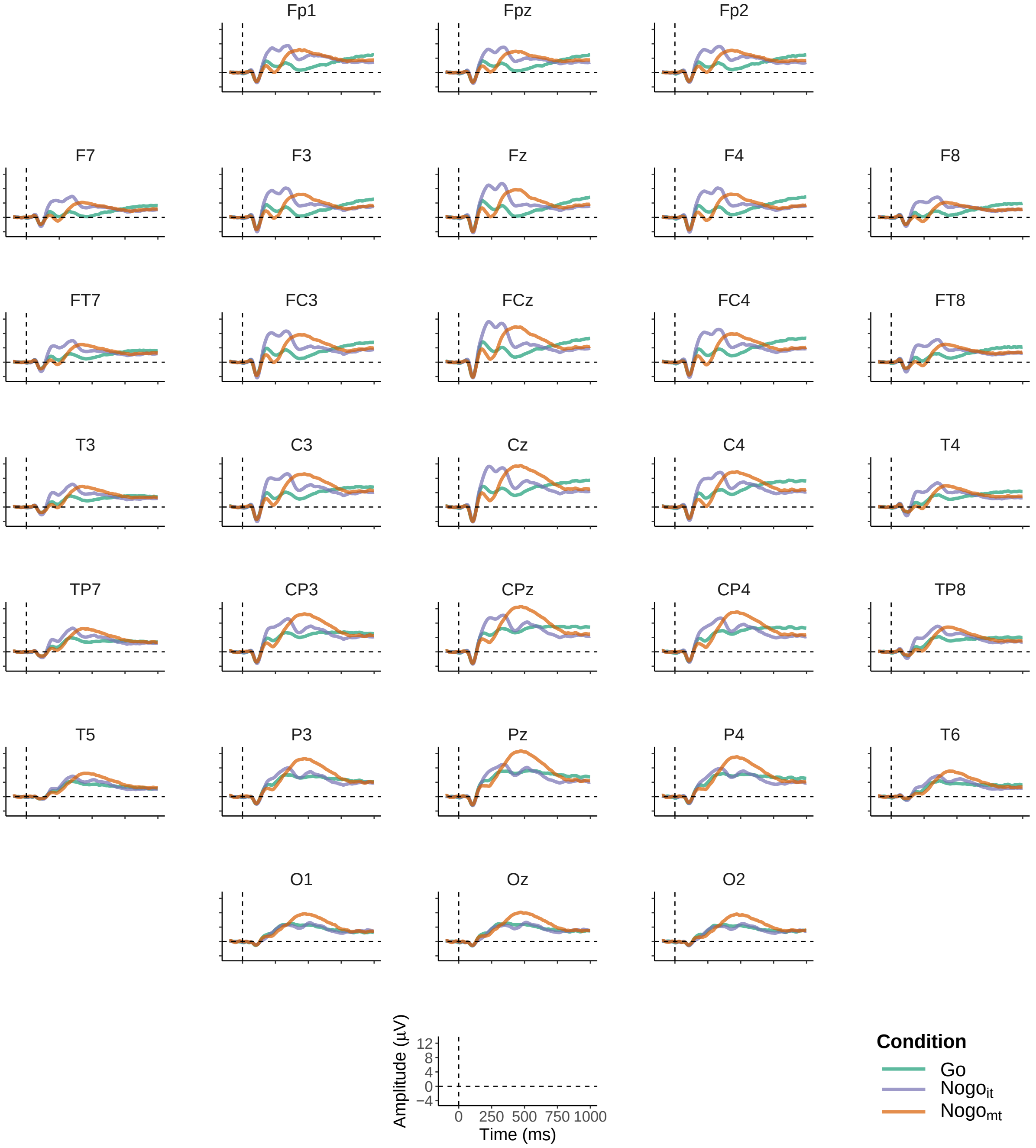

Supplementary Figure 1. Grand averages of the event-related potentials (ERPs) over all participants for the three cued conditions in the auditory Go/Nogo task (Go in green, Nogo$_{it}$ in violet, and Nogo$_{mt}$ in orange). Shown at all 31 electrodes.

Group comparison: Go

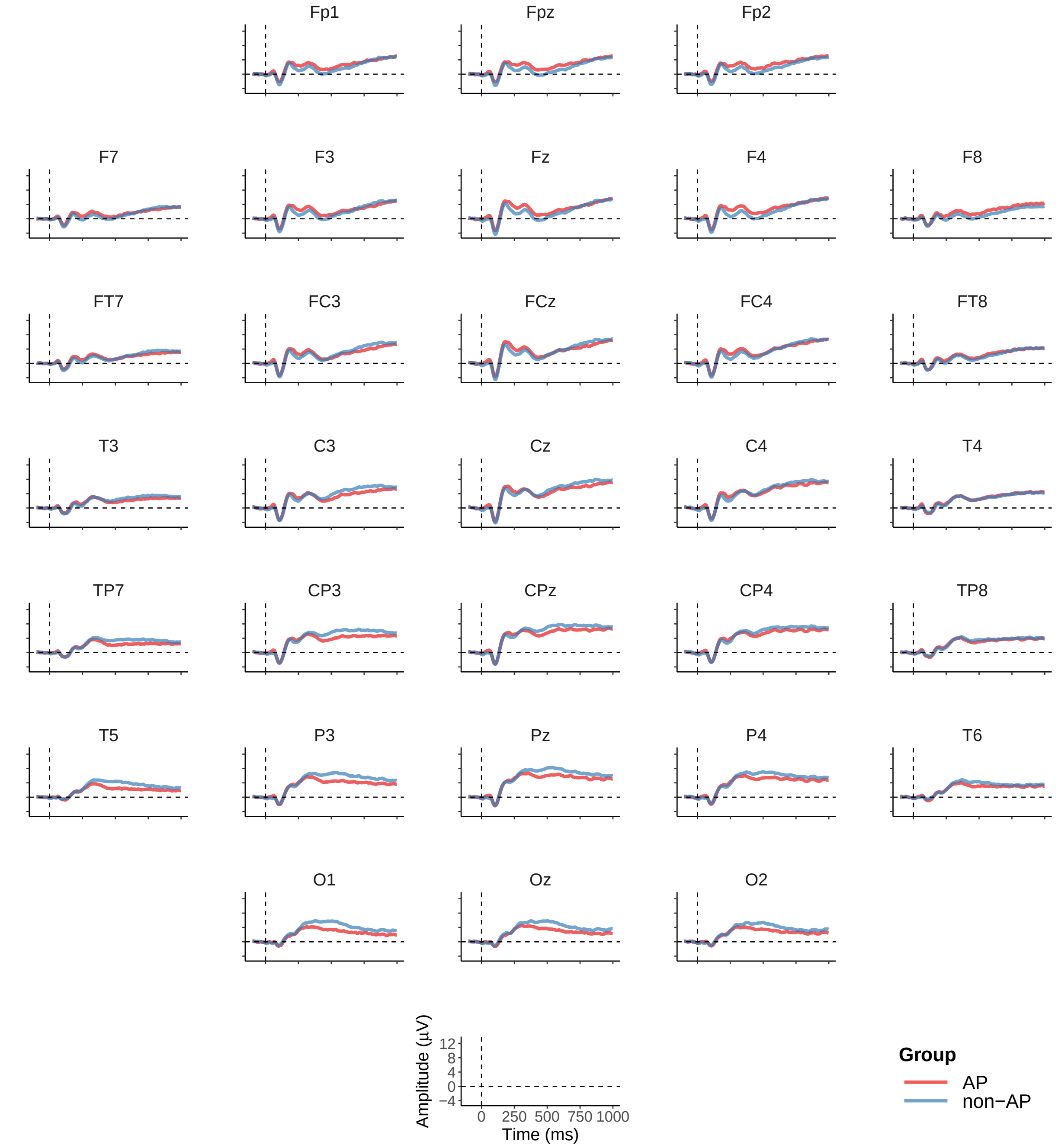

Supplementary Figure 2. Group-averaged ERPs for the Go condition at all 31 electrodes. Musicians with absolute pitch (AP) are shown in red, musicians without AP are shown in blue.

Group comparison: Nogo_{it}

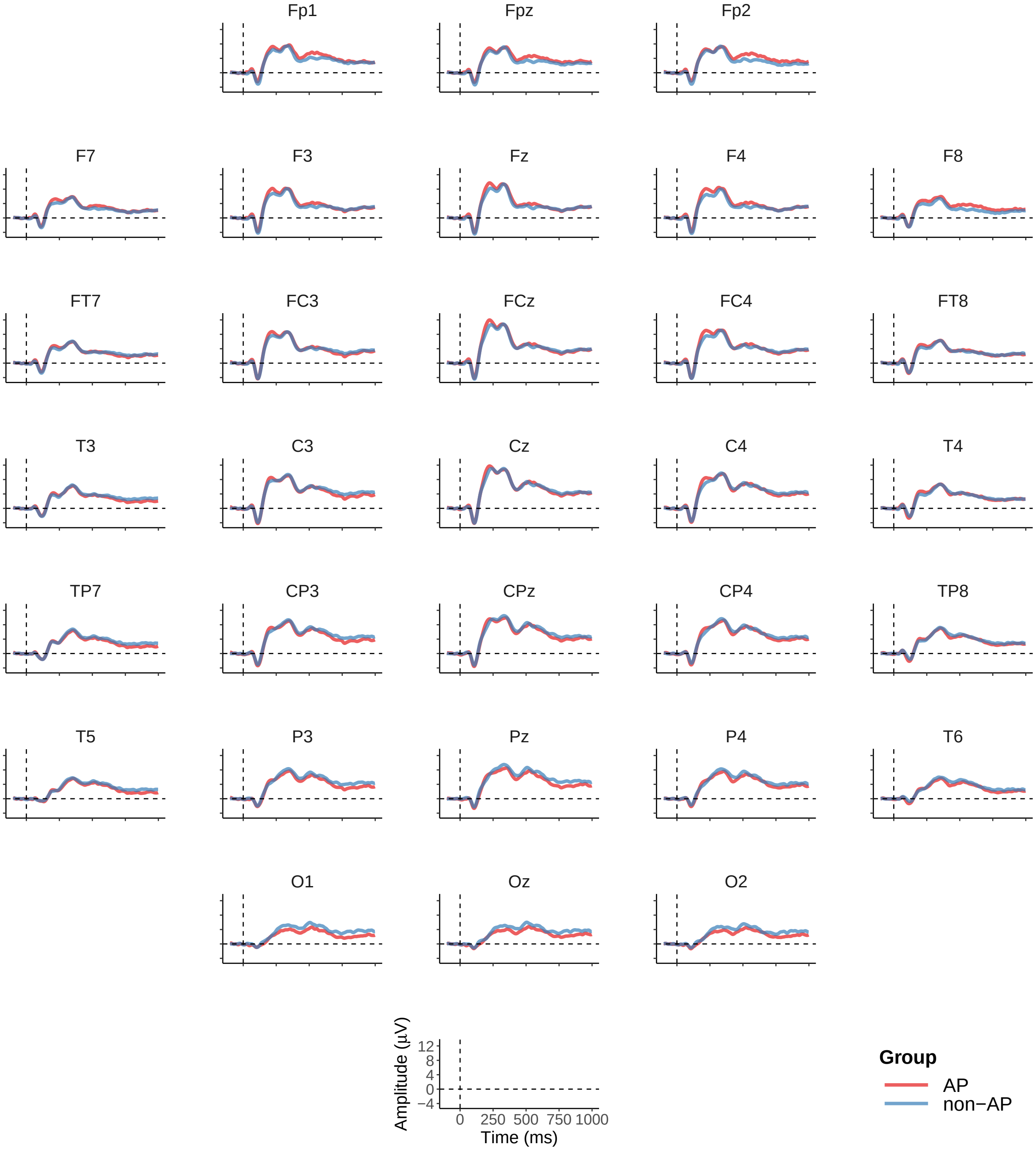

Supplementary Figure 3. Group-averaged ERPs for the Nogo_{it} condition at all 31 electrodes. Musicians with AP are shown in red, musicians without AP are shown in blue.

Group comparison: Nogo$_{mt}$

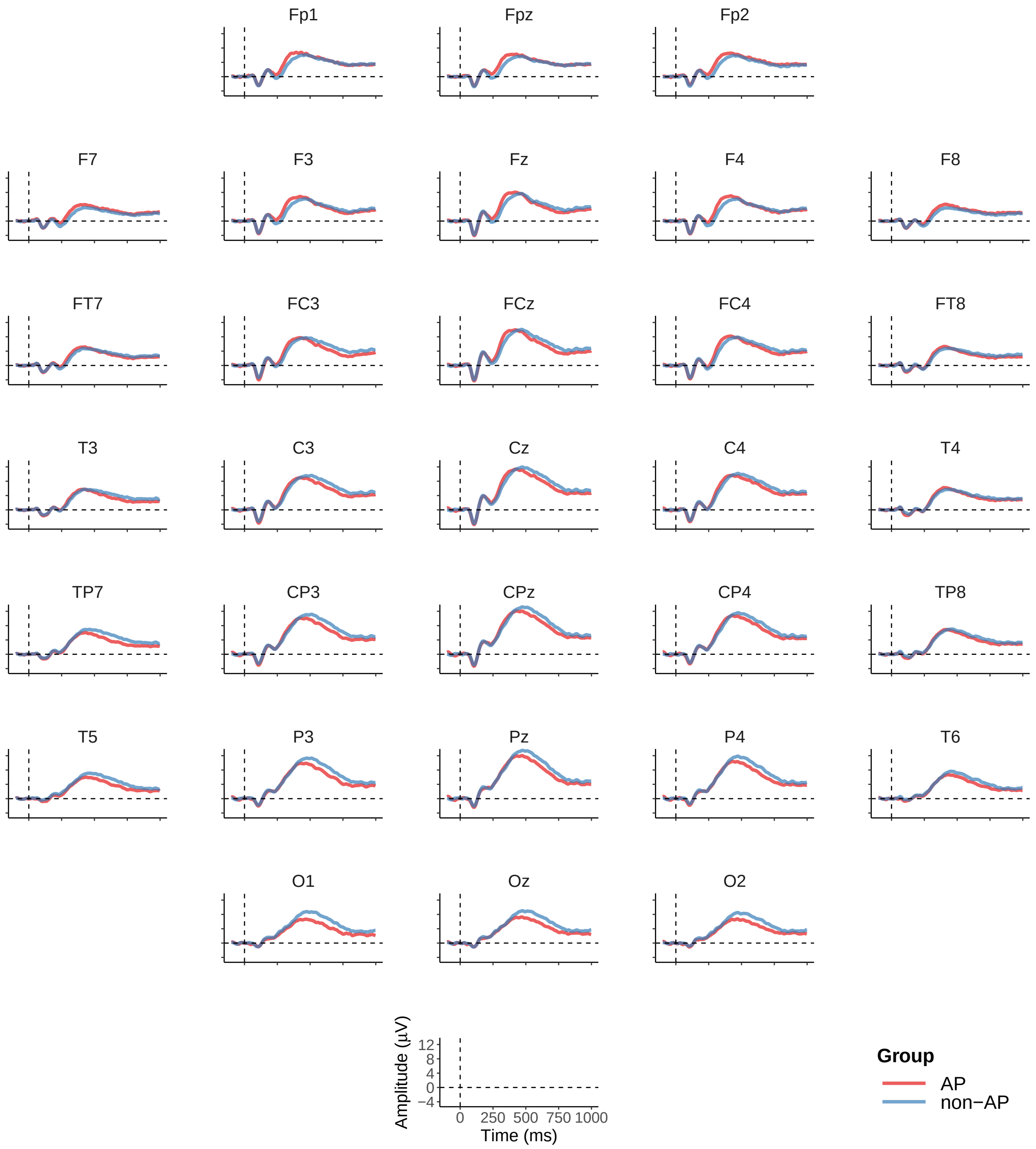

Supplementary Figure 4. Group-averaged ERPs for the Nogo$_{mt}$ condition separately for the two groups at all 31 electrodes. Musicians with AP are shown in red, musicians without AP are shown in blue.

Condition comparison: Nogo_{it}-Go, Nogo_{mt}-Go

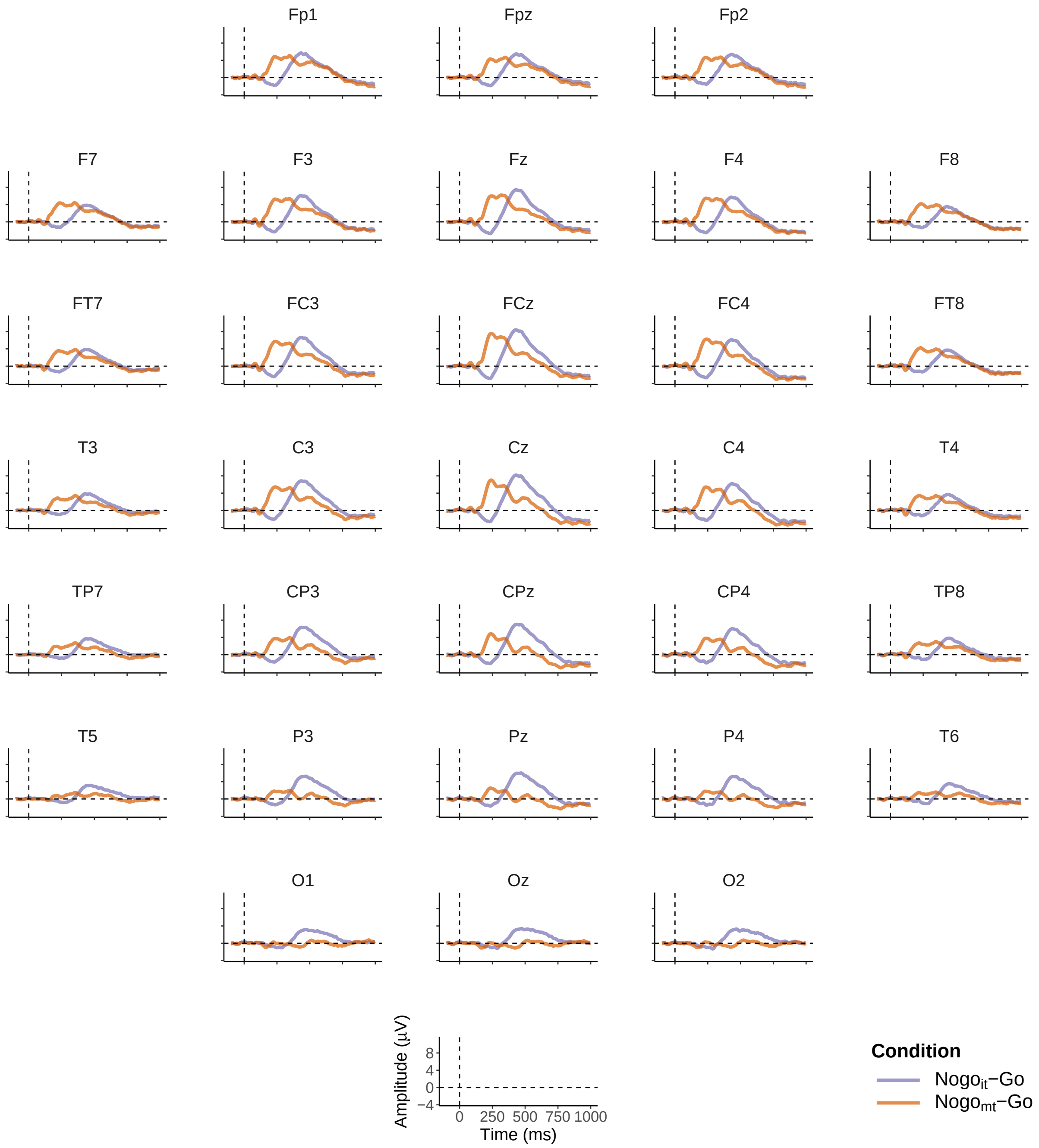

Supplementary Figure 5. Grand averages of the difference waves over all participants at all 31 electrodes. Nogo_{it}-Go is depicted in violet, Nogo_{mt}-Go is depicted in orange.

Group comparison: Nogo$_{it}$-Go

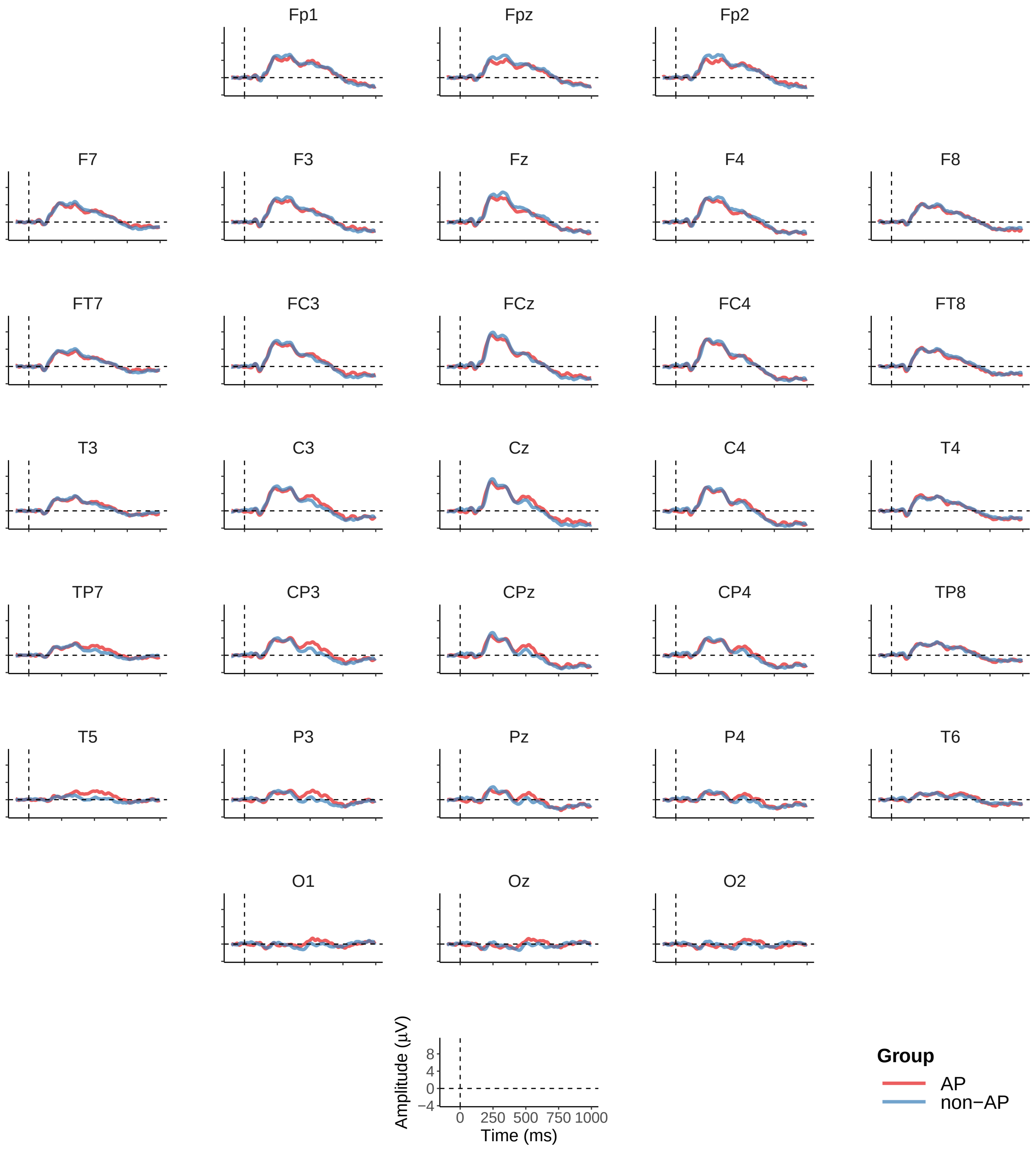

Supplementary Figure 6. Group averages of the difference wave Nogo$_{it}$-Go at all 31 electrodes. Musicians with AP are shown in red, musicians without AP are shown in blue.

Group comparison: Nogo$_{mt}$-Go

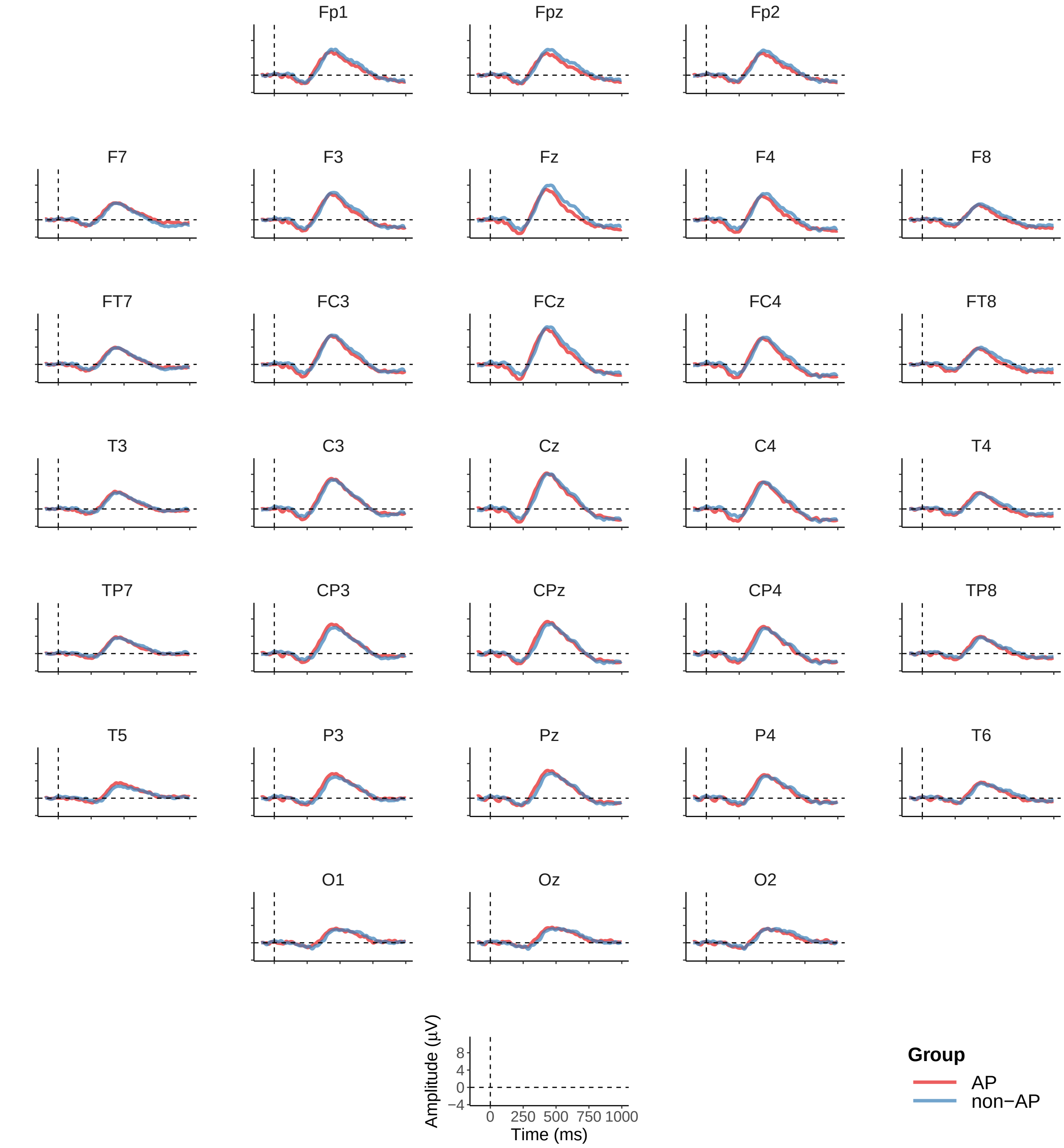

Supplementary Figure 7. Group averages of the difference wave Nogo$_{mt}$-Go at all 31 electrodes. Musicians with AP are shown in red, musicians without AP are shown in blue.
